# Evolution of Voltage-Dependent Anion Channel Function: From Molecular Sieve to Governator to Actuator of Ferroptosis

**DOI:** 10.3389/fonc.2017.00303

**Published:** 2017-12-19

**Authors:** John J. Lemasters

**Affiliations:** ^1^Center for Cell Death, Injury and Regeneration, Department of Drug Discovery and Biomedical Sciences, Medical University of South Carolina, Charleston, SC, United States; ^2^Department of Biochemistry and Molecular Biology, Medical University of South Carolina, Charleston, SC, United States

**Keywords:** erastin, ethanol, ferroptosis, reactive oxygen species, tubulin, voltage-dependent anion channel, Warburg phenomenon

## Abstract

The voltage-dependent anion channel (VDAC) is well known as the pathway for passive diffusion of anionic hydrophilic mitochondrial metabolites across the outer membrane, but a more complex functionality of the three isoforms of VDAC has emerged, as addressed in the *Frontiers in Oncology* Research Topic on “Uncovering the Function of the Mitochondrial Protein VDAC in Health and Disease: from Structure-Function to Novel Therapeutic Strategies.” VDAC as the single most abundant protein in mitochondrial outer membranes is typically involved in isoform-specific interactions of the mitochondrion with its surroundings as, for example, during mitochondria-dependent pathways of cell death. VDAC closure can also act as an adjustable limiter (governator) of global mitochondrial metabolism, as during hepatic ethanol metabolism to promote selective oxidation of membrane-permeant acetaldehyde. In cancer cells, high free tubulin inhibits VDAC1 and VDAC2, contributing to suppression of mitochondrial function in the Warburg phenomenon. Erastin, the canonical inducer of ferroptosis, opens VDAC in the presence of tubulin and hyperpolarizes mitochondria, leading to mitochondrial production of reactive oxygen species, mitochondrial dysfunction, and cell death. Our understanding of VDAC function continues to evolve.

In 1976, voltage-dependent anion channels (VDACs) were identified in mitochondria from *Paramecium tetraurelia* (1). Subsequent work, especially by Marco Colombini and his students, revealed VDAC to be a highly conserved ~30 kDa integral outer mitochondrial membrane protein that forms aqueous channels of ~3 nm diameter in the open state, allowing passage of molecules up to 5 kDa in size, although electrostatic profile is also an important determinant of channel permeance by charged molecules (2, 3). Consequently, VDAC is the common pathway for passive diffusion of all mostly anionic hydrophilic mitochondrial metabolites across the outer membrane. Thus, VDAC confers properties of a molecular sieve to the mitochondrial outer membrane, a long accepted and necessary attribute, but one that is, let’s face it, a little boring.

Positive and negative membrane potentials symmetrically close VDAC to most metabolites with half maximal closure at ±50 mV, indicating more complexity to VDAC functionality. With closure, pore diameter decreases to 1.8 nm, which blocks movement of respiratory substrates, adenine nucleotides, and other molecules that move in and out of mitochondria during metabolism (4). In its closed state, VDAC becomes a relatively cation selective channel that nonetheless conducts small anions like Cl^−^ (5). The question remains controversial whether voltage gating of VDAC is physiologically important, since Donnan potentials are too small for closure at the ionic strength of the intracellular milieu and because VDAC’s own permeability to small electrolytes collapses metabolism-driven potentials. However, metabolite channeling within complexes of hexokinase, creatine kinase, the adenine nucleotide translocator (ANT), and VDAC has been suggested to form a positive inside outer membrane potential sufficient to close VDAC (6, 7). Voltage gating may also be a fail-safe mechanism against loss of cellular metabolites should VDAC be inserted into the plasma membrane, as sometimes and somewhat controversially reported (8, 9), since cells have mechanisms to rapidly restore homeostasis of Na, K, Ca, and Cl ions passing through “closed” VDAC, whereas homeostatic restoration of anionic metabolites released through “open” VDAC occurs much more slowly.

Each of the three VDAC isoforms (VDAC1, VDAC2, and VDAC3) has very similar structure, conductance, and voltage-gating properties, but the existence of these different isoforms in humans and other mammalian species and their differential expression in various tissues suggests more functional complexity to VDAC (10–15). Since VDAC is the most abundant protein in the outer membrane, interactions of mitochondria with cytosolic proteins and other organelles typically involve VDAC, and generally speaking, these interactions are isoform specific. As well illustrated in this *Frontiers in Oncology* Research Topic on “Uncovering the Function of the Mitochondrial Protein VDAC in Health and Disease: from Structure-Function to Novel Therapeutic Strategies,” specific VDAC isoforms are important for pathways signaling both apoptotic and non-apoptotic cell death (16, 17). Moreover, Reina and coworkers review new evidence of a unique role for VDAC3 cysteine over-oxidation as a mitochondrial oxidative marker participating in reactive oxygen species (ROS) signaling (18).

Scientists studying VDAC have long wondered whether and how changes of VDAC conductance might regulate mitochondrial function. Concrete evidence for this came from studies showing closure of VDAC at an early stage of apoptosis (19), but what about VDAC in cells that are not dying? It was then proposed that various degrees of VDAC closing can act as a dynamic limiter, or “governator,” of global mitochondrial function (20). Such VDAC closure might account for unexplained anomalies of mitochondrial function seen in cells and tissues in certain contexts.

As an example, hepatic oxygen and ethanol metabolism nearly doubles within 2–3 h after gastric ethanol feeding, a so-called swift increase of alcohol metabolism (SIAM), which is an adaptive metabolic response to hasten the detoxification and elimination of both ethanol and its more toxic metabolite, acetaldehyde, by mostly alcohol dehydrogenase in the cytosol and aldehyde dehydrogenase in mitochondria (21, 22). Despite increased mitochondrial respiration, hepatic ATP paradoxically decreases by more than half after ethanol treatment but without activation of an identifiable ATPase. Moreover, steatosis occurs in parallel, indicative of inhibition of mitochondrial β-oxidation. To explain these phenomena, VDAC closure was proposed as a mechanism to inhibit mitochondrial release of ATP and uptake of fatty acyl-CoA, leading to cellular ATP depletion and steatosis, respectively. In addition, a protonophoric uncoupling pathway was hypothesized to open to stimulate respiration (20). Subsequent studies showed that the outer membranes of hepatocyte mitochondria do indeed become less permeable to adenine nucleotides and low molecular weight dextrans after ethanol and acetaldehyde treatment (23). Ethanol and acetaldehyde also decrease ureagenic respiration, a process requiring considerable flux of different metabolites through VDAC (24). Finally, intravital multiphoton microscopy reveals a reversible dose- and time-dependent depolarization of hepatocellular mitochondria after ethanol feeding in which depolarization occurs in an all-or-nothing fashion within any particular hepatocyte (25). Because small neutral aldehydes do not need VDAC or other carrier to cross the outer and inner membranes of mitochondria, VDAC closure and respiratory stimulation by uncoupling during SIAM together promote more rapid oxidation of membrane-permeant acetaldehyde while simultaneously inhibiting oxidation of competing substrates that require VDAC to enter mitochondria. These adaptations depend on hepatic aldehyde formation and revert as acetaldehyde is eliminated by oxidation to acetate. Similar mechanisms may also play a role in nonalcoholic steatohepatitis in which oxidative stress is an important component, since lipid peroxidation chain reactions generate aldehydes like malondialdehyde that close VDAC even more potently than acetaldehyde (24, 26).

Another example of a possible governator role for VDAC is the suppression of mitochondrial metabolism and enhancement of aerobic glycolysis that characterize the pro-proliferative Warburg metabolic phenotype of cancer cells. In this hypothesis, VDAC closure decreases mitochondrial ATP release, lowers cytosolic ATP/ADP, and thereby stimulates glycolysis (20). Hexokinase may be one molecule inhibiting VDAC, since hexokinase is overexpressed in cancer cells and inhibits VDAC reconstituted into planar lipid bilayers (27).

A more important VDAC closer in cancer cells is free dimeric tubulin, as reviewed in the Research Topic by Maldonado (28). Proliferating cancer cells have much higher levels of free tubulin compared to post-mitotic cells, because tubulin is needed for spindle formation at metaphase. Rostovtseva’s group showed that low nanomolar free tubulin closes VDAC reconstituted into planar bilayers (29). VDAC1 and VDAC2, the most abundant isoforms in most cells, are sensitive to tubulin, whereas the least abundant VDAC3 is insensitive (14). In intact cancer cells, increased free tubulin after microtubule depolymerization causes mitochondrial membrane potential (ΔΨ, an indicator of mitochondrial metabolism) to decrease, apparently due to the inhibition of respiratory substrate entry through VDAC (30). By contrast, microtubule stabilization with paclitaxel, which decreases free tubulin, causes ΔΨ to increase. Remarkably, the VDAC-binding molecule erastin blocks the inhibitory effect of free tubulin on VDAC inserted into lipid bilayers and reverses mitochondrial depolarization induced by elevated free dimeric tubulin in intact cancer cells (14, 31).

Erastin is the canonical inducer of ferroptosis, a type of non-apoptotic, oxidative cell death that is so-named because the iron chelator, desferal (deferoxamine), prevents erastin-induced cell killing (32). Desferal is well known to protect against many oxidative stresses, including ischemia-reperfusion, drug-induced hepatotoxicity, and exposure to oxidant chemicals (33–37). How then can VDAC opening be linked to oxidative stress? The answer is that mitochondrial ROS generation increases with increasing mitochondrial ΔΨ and with increased reduction of the respiratory chain as respiratory substrates enter following VDAC opening (38–42). Thus, erastin and several erastin-like small molecules identified by high content screening increase mitochondrial ROS in parallel with mitochondrial hyperpolarization, as highlighted in Maldonado’s review (28, 43, 44)^1^. At later time points, ROS-dependent mitochondrial dysfunction occurs, which likely represents onset of the mitochondrial permeability transition. Ultimately cell death occurs, which is prevented by antioxidants like *N*-acetylcysteine. Thus, VDAC opening is what Maldonado calls an “anti-Warburg pro-oxidant switch” that leads to reversion of the pro-proliferative Warburg metabolic phenotype of aerobic glycolysis, increased ΔΨ, mitochondrial ROS generation, and oxidative stress-induced ferroptoic cell death.

Overall, our understanding of VDAC function continues to evolve. No longer just a sieve allowing passive movement of small molecules across the mitochondrial outer membrane, VDAC is an active agent regulating interactions between mitochondria and their surroundings, global mitochondrial metabolism, and the life or death fate of cells. Not so boring after all.

## Author Contributions

JJL is responsible for the content of this article.

## Conflict of Interest Statement

The author declares that the research was conducted in the absence of any commercial or financial relationships that could be construed as a potential conflict of interest.

## Footnotes

^1^deHart DN, Fang D, Heslop K, Li L, Lemasters JJ, Maldonado EN. Opening of voltage dependent anion channels promotes reactive oxygen species generation, mitochondrial dysfunction and cell death in cancer cells. (2017) Submitted for publication.
